# Regulation of Carbohydrate-Responsive Metabolic Genes by Histone Acetylation and the Acetylated Histone Reader BRD4 in the Gene Body Region

**DOI:** 10.3389/fmolb.2021.682696

**Published:** 2021-07-15

**Authors:** Kazuki Mochizuki, Shiori Ishiyama, Natsuyo Hariya, Toshinao Goda

**Affiliations:** ^1^ Department of Local Produce and Food Sciences, Faculty of Life and Environmental Sciences, University of Yamanashi, Yamanashi, Japan; ^2^ Department of Integrated Applied Life Science, Integrated Graduate School of Medicine, Engineering, and Agricultural Sciences, University of Yamanashi, Yamanashi, Japan; ^3^ Department of Nutrition, Faculty of Health and Nutrition, Yamanashi Gakuin University, Yamanashi, Japan; ^4^ Graduate School of Nutritional and Environmental Sciences, University of Shizuoka, Shizuoka, Japan

**Keywords:** BRD4, carbohydrate, gene body-epigenetics, histone acetylation, metabolic diseases, transcriptional elongation reaction, type 2 diabetes

## Abstract

Studies indicate that induction of metabolic gene expression by nutrient intake, and in response to subsequently secreted hormones, is regulated by transcription factors binding to cis-elements and associated changes of epigenetic memories (histone modifications and DNA methylation) located in promoter and enhancer regions. Carbohydrate intake-mediated induction of metabolic gene expression is regulated by histone acetylation and the histone acetylation reader bromodomain-containing protein 4 (BRD4) on the gene body region, which corresponds to the transcribed region of the gene. In this review, we introduce carbohydrate-responsive metabolic gene regulation by (i) transcription factors and epigenetic memory in promoter/enhancer regions (promoter/enhancer-based epigenetics), and (ii) histone acetylation and BRD4 in the gene body region (gene body-based epigenetics). Expression of carbohydrate-responsive metabolic genes related to nutrient digestion and absorption, fat synthesis, inflammation in the small intestine, liver and white adipose tissue, and in monocytic/macrophage-like cells are regulated by various transcription factors. The expression of these metabolic genes are also regulated by transcription elongation *via* histone acetylation and BRD4 in the gene body region. Additionally, the expression of genes related to fat synthesis, and the levels of acetylated histones and BRD4 in fat synthesis-related genes, are downregulated in white adipocytes under insulin resistant and/or diabetic conditions. In contrast, expression of carbohydrate-responsive metabolic genes and/or histone acetylation and BRD4 binding in the gene body region of these genes, are upregulated in the small intestine, liver, and peripheral leukocytes (innate leukocytes) under insulin resistant and/or diabetic conditions. In conclusion, histone acetylation and BRD4 binding in the gene body region as well as transcription factor binding in promoter/enhancer regions regulate the expression of carbohydrate-responsive metabolic genes in many metabolic organs. Insulin resistant and diabetic conditions induce the development of metabolic diseases, including type 2 diabetes, by reducing the expression of BRD4-targeted carbohydrate-responsive metabolic genes in white adipose tissue and by inducing the expression of BRD4-targeted carbohydrate-responsive metabolic genes in the liver, small intestine, and innate leukocytes including monocytes/macrophages and neutrophils.

## Introduction

Excessive carbohydrate intake, in particular digested carbohydrates, and excessive intake of other energy nutrients including fat and protein, leads to the development and progression of life-style diseases including obesity, type 2 diabetes, and related complications. Carbohydrate intake and/or glucose signals enhance the expression of many metabolic genes related to carbohydrate digestion and absorption in the small intestine, those related to fat synthesis in many metabolic organs including the liver and adipose tissue, and those related to inflammation in innate leukocytes such as neutrophils and monocytes/macrophages. Induction of metabolic gene expression by carbohydrate intake, and in response to subsequently secreted hormones such as insulin, is regulated by transcription factors that bind to *cis*-elements located in promoter and enhancer regions. Such transcription factors include carbohydrate response element binding protein (ChREBP) (Ortega-Prieto and Postic, 2019), sterol regulatory element-binding transcription factor 1 (SREBF1) (Eberle et al., 2004), and nuclear factor-kappa B (NF-κB) (Tan et al., 2018). Regulation within promoter and enhancer regions is based on transcription initiation, which involves the recruitment of RNA polymerase II to the promoter region. Recent studies have shown that carbohydrate intake-mediated induction of many metabolic genes is regulated by histone acetylation and the histone acetylation reader bromodomain-containing protein 4 (BRD4) on the gene body region, which corresponds to the transcribed region of the gene (Honma et al., 2007, 2009, 2013; Honma et al., 2014; Inamochi et al., 2016; Yamada et al., 2016; Sakurai et al., 2017; Yamauchi et al., 2018; Inoue et al., 2019; Mochizuki et al., 2020). BRD4 belongs to the bromodomain and extraterminal (BET) protein family with two tandem bromodomains that interact with acetylated histones (Taniguchi, 2016). BRD4 promotes the transcription elongation step by enhancing RNA polymerase II phosphorylation at serine two in the C-terminal domain (Devaiah et al., 2012) and/or by recruiting positive transcription elongation factor b (P-TEFb) upon histone acetylation (Jang et al., 2005). BRD4 enhances the transcriptional elongation reaction by recruiting complexes to the proximal promoter region and acetylated histones in the gene body (Kanno et al., 2014). The transcription elongation reaction, which is stimulated by histone acetylation and BRD4 binding to acetylated histones on the gene body and/or proximal promoter region, and the transcription initiation reaction are triggered by transcription factors, and these reactions are essential for expression of metabolic genes. BRD4 and histone acetylation of carbohydrate-responsive metabolic genes are found in the gene body region rather than in promoter/enhancer regions (Honma et al., 2007, 2009, 2013; Honma et al., 2014; Inamochi et al., 2016; Yamada et al., 2016; Sakurai et al., 2017; Yamauchi et al., 2018; Inoue et al., 2019; Mochizuki et al., 2020). Here, we review general carbohydrate-responsive transcriptional regulation by transcription factors, and novel carbohydrate-responsive gene body epigenetic mechanisms by acetylation and the histone acetylation reader, BRD4. In addition, we discuss their involvement in the development of metabolic diseases through disruption of gene body epigenetics.

### Induction of Metabolic Genes by Carbohydrate Intake

**FIGURE 1:**
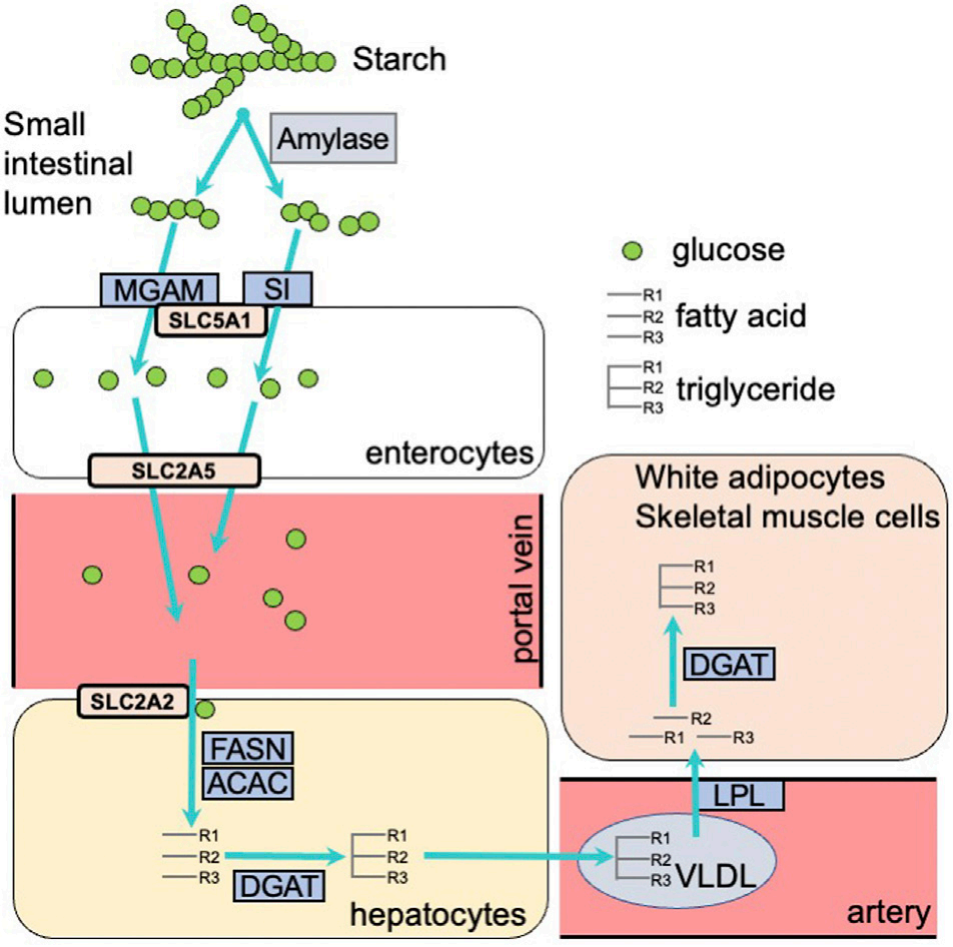
Carbohydrate digestion and absorption in the small intestine, and carbohydrate metabolism in the liver and white adipose tissue. MGAM, maltase-glucoamylase; SI, sucrase-isomaltase; SLC5A1, solute carrier family 5 member 1; SLC2A5, solute carrier family 2 member 5; SLC2A2, solute carrier family 2 member 2; FASN, fatty acid synthase; ACAC, acetyl-CoA carboxylase; DGAT, diacylglycerol *O*-acyltransferase; VLDL, very low density lipoprotein; LPL, lipoprotein lipase.

**FIGURE 2:**
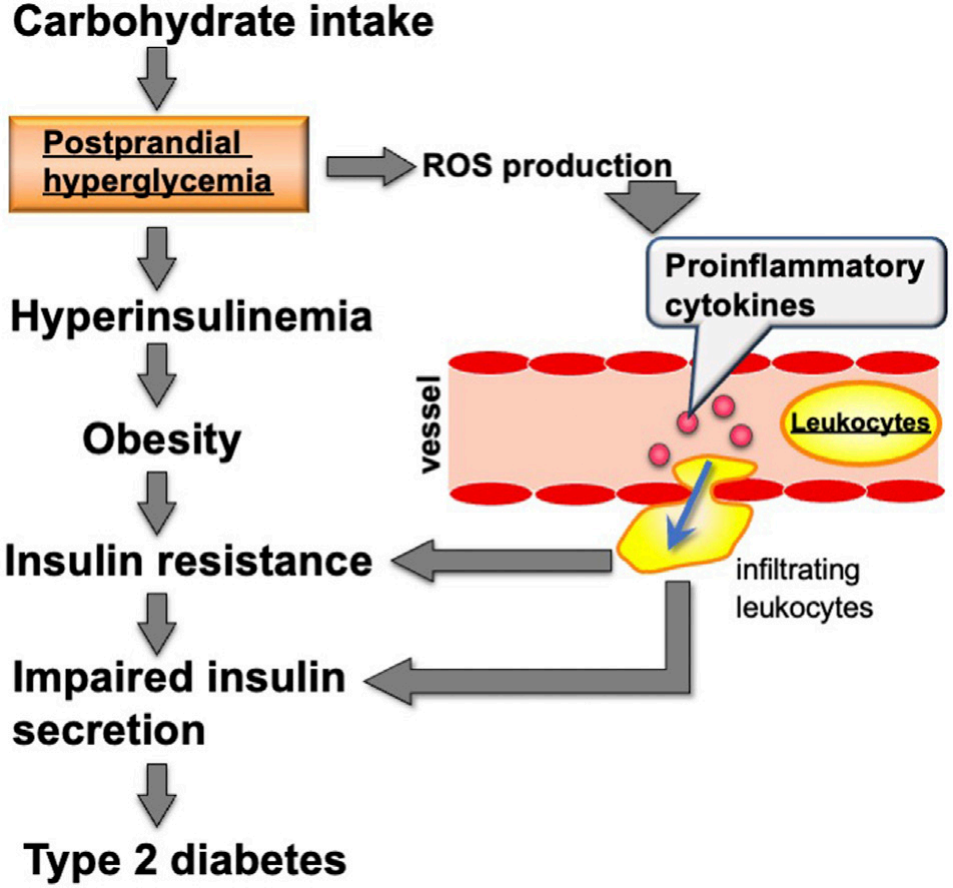
Relationship between carbohydrate intake and metabolic diseases including type 2 diabetes. ROS: reactive oxygen species.

Carbohydrate intake induces the expression of many metabolic genes. This gene expression contributes to carbohydrate digestion in the gastrointestinal tract and enhances the activity of the glycolytic pathway and fatty acid synthesis in metabolic organs including the liver, white adipose tissue, and skeletal muscle. After carbohydrate intake, *α*-amylase in saliva and pancreatic fluid and disaccharidases such as sucrase-isomaltase (SI) and maltase-glucoamylase (MGAM) on enterocytes of the small intestine facilitate carbohydrate digestion. Subsequently, hexoses (glucose, fructose, and galactose), which are products from the digestion of starch and sucrose, are incorporated into the enterocytes of the small intestine using transporters including the glucose/galactose transporter solute carrier family five member 1 (SLC5A1) and the fructose transporter solute carrier family two member 5 (SLC2A5) (Gray, 1992). Several studies demonstrated that *Si*, *Mgam*, *Slc5a1*, and *Slc2a5* expression is induced in the small intestine of rodents by feeding them carbohydrates including starch, sucrose, or fructose (Mochizuki et al., 2010; Yoshinaga et al., 2012; Inoue et al., 2015). These hexoses, where most fructose and galactose are converted to glucose, are incorporated into cells of metabolic organs, including the liver, skeletal muscle, and white adipose tissue. Glucose is transported into the liver by the SLC2A2 transporter, which transports glucose into cells by facilitated diffusion. Glucose is transported into white adipose tissue in an insulin-dependent manner *via* SLC2A4 (Chadt and Al-Hasani, 2020). In liver and white adipose tissue cells, glucose is converted into triacylglycerol *via* glycolysis, fatty acid synthesis, and triacylglycerol synthesis pathways. Triacylglycerols synthesized in the liver are transported to many organs, including white adipose tissue and skeletal muscle, by very low density lipoprotein (VLDL). The rate-controlling enzyme of triacylglycerol transport from VLDL to adipose tissue and skeletal muscle is lipoprotein lipase (LPL). Fatty acids digested by LPL are incorporated into cells and re-synthesized to triacylglycerol by diglyceride acyltransferase (DGAT) (Figure 1). The cascades from glucose incorporation to fat synthesis and fat accumulation in white adipose tissue and skeletal muscle involve the expression of many genes. In rodents, carbohydrate intake and/or insulin secretion upregulate the expression of *Slc2a4* in adipose tissue and skeletal muscle (Zanquetta et al., 2014), glycolytic-related genes like pyruvate kinase (*Pklr*), fatty acid synthesis-related genes such as fatty acid synthase (*Fasn*) and acetyl-CoA carboxylases (*Acaca* and *Acacb*), and the triacylglycerol synthesis-related genes *Lpl* and *Dgat* (Ranganathan et al., 2006; Iizuka, 2017). Postprandial hyperglycemia observed after carbohydrate intake induces pro-inflammatory cytokine expression, including interleukin 1 beta (*Il1b*) and tumor necrosis factor-α (*Tnfa*) in peripheral leukocytes (Tanaka et al., 2009). Excessive carbohydrate intake induces type 2 diabetes by enhancing expression of genes related to triglyceride synthesis in the liver and adipose tissue, and the expression of genes related to inflammation in innate leukocytes (Figure 2).

### Regulation of Carbohydrate Responsible Genes by Transcriptional Factors

Carbohydrate digestion/absorption-related genes, such as *Si* and *Mgam*, in the small intestine of rodents are regulated by homeobox transcription factors including caudal-type homeobox 2 (CDX2) and hepatocyte nuclear factor 1 (HNF1). Here, carbohydrate intake enhances the binding of CDX2 and HNF1 to the promoter regions of *Si* and *Mgam* (Honma et al., 2007; Mochizuki et al., 2010). The expression of glycolysis-related target genes including *Slc2a2* and *Pklr* in the liver, *Slc2a5* in the small intestine, and lipogenesis-related genes (*Acaca*, *Acacb*, and *Fasn*) in the liver and adipose tissue (Iizuka, 2017) are enhanced by the transcription factor ChREBP. Insulin-dependent expression of lipogenesis related genes such as *Acaca*, *Acacb*, and *Fasn* are regulated by sterol regulatory element-binding protein 1c (SREBF1c) (Shimano, 2001).

In innate leukocytes, high glucose and associated reactive oxygen species (ROS) production in THP-1 monocyte/macrophage-like cells were shown to enhance *IL1B* expression by NF-κB (Jo et al., 2020). Taken together, these data show that there are many transcription factors that respond to carbohydrate ingestion.

### Epigenetic Regulation in Promoter/Enhancer- and Gene Body-Regions

**FIGURE 3:**
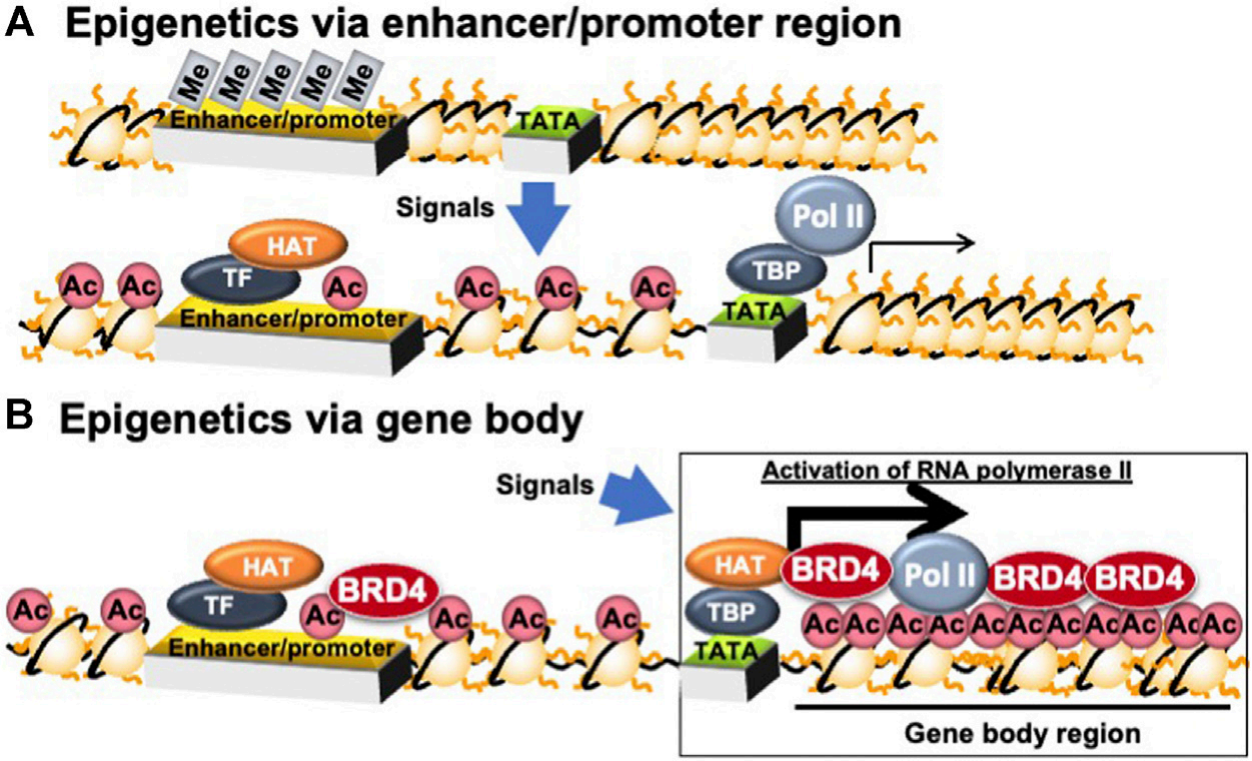
Epigenetic regulation in enhancer/promoter- and gene body-regions. Ac, histone acetylation; BRD4, bromodomain containing 4; HAT, histone acetyl-transferase; Me, DNA methylation; Pol II, RNA polymerase II; TATA, TATA box; TBP, TATA box binding protein; TF, transcription factor.

Epigenetic memory can be classified as DNA methylation or histone modification. DNA methylation involves adding methyl group to cytosine nucleotides within DNA. Hypermethylation of cytosine nucleotides in CG repeat sequences (CpG islands) in and around genes induces heterochromatin formation and represses gene transcription by association with methylated DNA binding proteins. Typical histone modifications include acetylation, methylation, phosphorylation, and ubiquitination (Fuks, 2005). Histone acetylation relaxes the chromatin and activates transcription by changing the chromatin charge and recruiting proteins for transactivation (Bruce Alberts et al., 2015). Histone acetyl-transferase (HAT) enhances transcription by acetylating histones and recruiting bromodomain proteins, which bind to acetylated histones and transcription initiation complexes in promoter and enhancer regions (Figure 3A).

**FIGURE 4:**
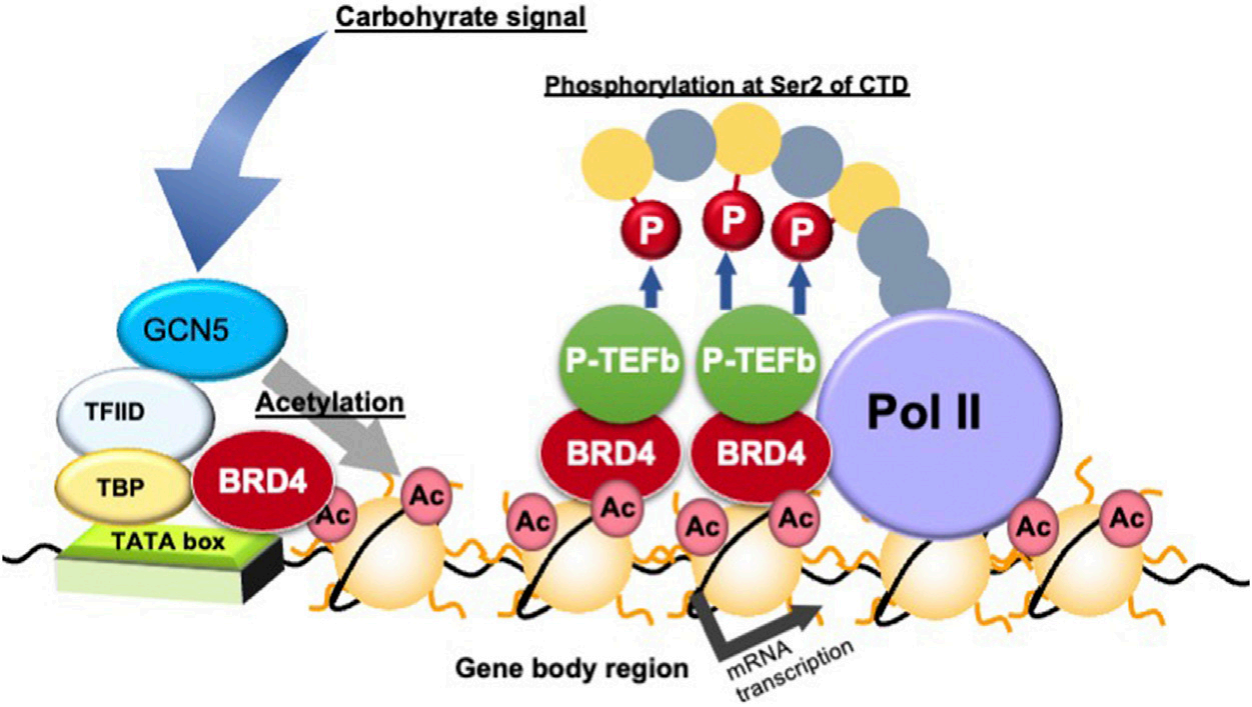
Transcription elongation process *via* gene body epigenetics. Ac, histone acetylation; BRD4, bromodomain containing 4; GCN5, general control of amino acid synthesis 5; P-TEFb, positive elongation factor b; P, phosphorylation; Pol, RNA polymerase II; TATA, TATA box; TBP, TATA box binding protein; Ser, serine residue; TFIID, transcription factor IID.

BRD4 binds to acetylated histones on the gene body and proximal promoter regions to enhance transcription elongation by phosphorylating the second serine residue of the C-terminal domain (CTD) of RNA polymerase II and/or recruiting P-TEFb, a cyclin T1-CDK9 complex (Jang et al., 2005) (Figure 4). Devaiah et al. reported that BRD4 phosphorylates the second serine residue of the CTD of RNA polymerase II without P-TEFb (Devaiah et al., 2012). BRD4 regulates transcription elongation reactions by two mechanisms: (i) inducing the recruitment of the elongation complex on the proximal promoter region; and (ii) inducing the binding of the transcription elongation complex on acetylated histones in the gene body region (Kanno et al., 2014). The former regulation mechanism by BRD4 is independent of histone acetylation, whereas the later regulation mechanism is dependent on histone acetylation (Kanno et al., 2014). BRD4 regulates the rate of cell cycle-related gene mRNA synthesis during the middle of the G1 phase, which is when the mRNA of genes related to G1-S progression is increasing rapidly (Jang et al., 2005; Mochizuki et al., 2008). Furthermore, inactivation of BRD4 activity or downregulation of BRD4 expression by inhibitors is used in cancer therapy target to repress these cell cycle-related genes (Zhang et al., 2021).

### Epigenetic Regulation in Gene Body Regions by Carbohydrate Signals

Honma et al. demonstrated that feeding rats a diet high in starch, the main source of carbohydrates for humans, for a week enhanced histone acetylation in gene body- and promoter/enhancer-regions and mRNA expression of carbohydrate digestion/absorption-related genes including *Si* and *Sglt1* (Honma et al., 2007; 2009; 2013). Furthermore, a high-starch diet was found to enhance general control of amino acid synthesis 5 (GCN5; a HAT) binding and acetylation of histone H3 and H4 around the gene body- and promoter/enhancer-regions of *Si* and *Slc5a1* and their expression (Inoue et al., 2011). Treatment of intestinal Caco-2 cells with dexamethasone, a glucocorticoid agonist with p44/42 mitogen-activated protein kinase inhibition properties, was found to enhance *Slc2a5* and BRD4 binding and histone acetylation on both promoter/enhancer and gene body regions of the gene (Inamochi et al., 2016). Houzelstein et al. reported that a heterogenic deficiency of *Brd4* reduces adipose tissue weight (Houzelstein et al., 2002). Inamochi et al. demonstrated that *Brd4* heterozygous mice show lower *Slc2a5* expression in the small intestine (Inamochi et al., 2016). Furthermore, re-feeding rats a high-sucrose diet after 3 days of starvation enhanced histone H3 acetylation in the *Slc2a5* gene body and increased *Slc2a5* mRNA expression (Honma et al., 2014). The circadian rhythmic expression of *Slc5a1* in the small intestine of mice fed a regular high carbohydrate diet is regulated by histone acetylation and binding of BRD4-P-TEFb to the gene body region of the gene (Yamauchi et al., 2018). Thus, many genes related to carbohydrate digestion/absorption in the small intestine in response to carbohydrate intake are regulated by histone acetylation and BRD4. In particular, gene body epigenetics are closely related to regulation of genes involved in carbohydrate metabolism.

Yamada et al. demonstrated that force-feeding mice fructose induces the expression of many lipid accumulation-related genes including *Cyp8b1*, *Dak*, and *Plin5* as well as BRD4 binding and histone acetylation on the gene body and promoter/enhancer regions of those genes in mouse liver (Yamada et al., 2016). In mouse white adipocyte-like cells, the expression of insulin sensitivity genes is downregulated by *Brd4* shRNA and/or the (+)-JQ1 BET family inhibitor, and among them, expression of *Adipoq*, *Glut4*, *Lpl*, *Dgat2*, and *Gpd1*, are regulated by BRD4 and the acetylated gene body and promoter/enhancer region histones (Sakurai et al., 2017; Inoue et al., 2019; Mochizuki et al., 2020).

In leukocytes, LPS stimulation in mice lymphocytes has been demonstrated to regulate the amount of BRD4 (Dey et al., 2000). Additionally, induction of *TNFA* in human monocyte/macrophage-like THP-1 cells exposed to high glucose concentrations and/or TNFA is associated with an increase in acetylation of gene body histones (Honma et al., 2020).

Histone H3 acetylation and P-TEFb interaction with this histone for genes mentioned above occur more often in the gene body when compared with that of promoter/enhancer regions, and acetylation in the gene body are induced by signals, including carbohydrates and cell differentiation. In addition, carbohydrate intake induces histone acetylation of the gene body region rather than transcription factor binding, e.g., CDX2 and HNF1, and this acetylation has been observed for carbohydrate digestion/absorption-related genes such as *Si* and *Sglt1* in the small intestine of mice and rats (Honma et al., 2007; Inoue et al., 2011). In addition, histone H3 acetylation rather than transcription factor bindings of ChREBP, for example, in the vicinity of *Fasn* in the liver of SHR/NDmc-cp rats is closely and positively associated with *Fasn* mRNA (Suzuki et al., 2015). In addition, induction of histone H3 acetylation around *Adipoq* during 3T3-L1 adipocyte differentiation is greater than PPARγ transcription factor binding in the proximity of the gene (Sakurai et al., 2017). Therefore, induction of many carbohydrate-responsive genes is regulated by histone acetylation around those genes, in particular in the gene body region, rather than the binding of transcription factors.

Histone acetylation and BRD4 binding in promoter/enhancer regions are also enhanced by carbohydrate signals or the differentiation signals mentioned above. Kanno et al. demonstrated that BRD4 with P-TEFb include a proximal promoter and a gene body region (Kanno et al., 2014). In addition, BRD4 was demonstrated to bind the mediator complex, which mediates the formation of the transcription initiation complex by binding to transcription factors, including thyroid hormone receptor associated protein 220 (TRAP220) (Jang et al., 2005). In addition, Sakurai et al. demonstrated that TRAP220 is recruited to a carbohydrate responsive metabolic gene *Adipoq* in 3T3-L1 adipocytes (Sakurai et al., 2017). Based on gene body epigenetics involving histone and proximal promoter region acetylation, BRD4 regulates the transcription elongation process. In addition, the transcription initiation reaction is triggered by binding of transcription factors, which are essential for regulating the expression of metabolic genes.

### Disruption of Epigenetic Regulation in the Gene Body Region and Development of Metabolic Diseases

**FIGURE 5:**
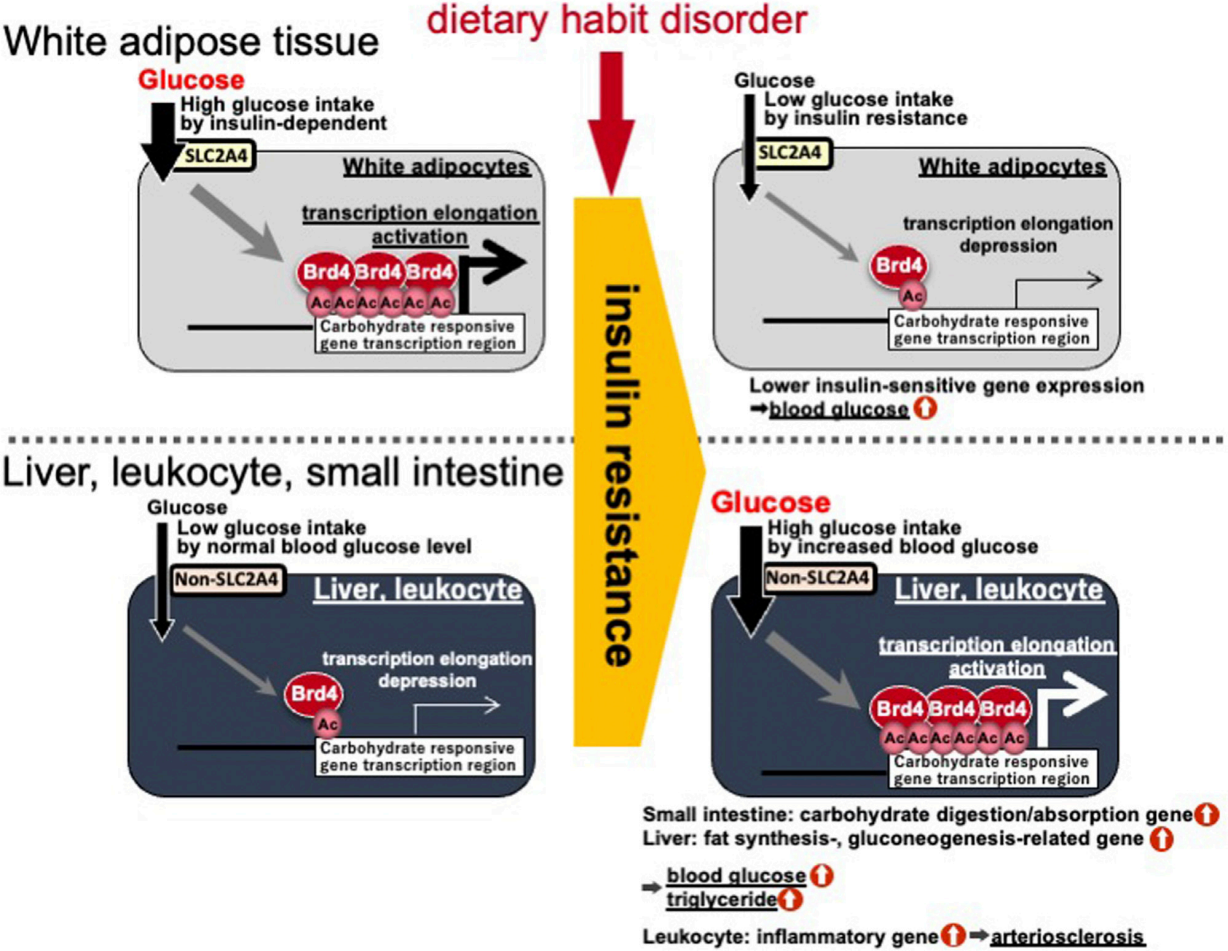
Differences in gene body epigenetics in white adipocytes and other tissues (liver, monocytic cells, and small intestine) in the insulin resistant/diabetic state. Ac, histone acetylation; BRD4, bromodomain containing 4; SLC2A4, solute carrier family 2 member 4.

Gene body epigenetics *via* histone acetylation and BRD4 binding are closely involved in lipid accumulation after meals by responding to carbohydrate- and insulin-signals. Treatment of mice with the (+)-JQ1 inhibitor (a potent inhibitor of the BET family) caused a reduction in the average mouse body weight and suppressed the expression of fibroblast growth factor (FGF)-15, which inhibits gluconeogenesis, induces fatty acid oxidation, and leads to higher glucose concentrations in the liver (Kozuka et al., 2019). In addition, *Brd4* (+/−) mice are lean (Houzelstein et al., 2002). In this study, BRD4 targets many of the lipid synthesis genes mentioned above. Therefore, deficiency and inhibition of BRD4 may induce type 2 diabetes. Insulin resistance in obese people is a critical step toward developing type 2 diabetes. Treatment with the TNFA insulin resistant inducer reduces histone acetylation and BRD4 binding in the gene body region of adiponectin (*Adipoq*), and *Lpl* in white adipocytes (Sakurai et al., 2009; Inoue et al., 2019). Conversely, insulin resistance and/or diabetic states in the liver of SHR/NDmc-cp rats, which are models of metabolic syndrome, was found to enhance histone acetylation in the gene body region of *Fasn* and *Fasn* gene expression (Suzuki et al., 2015). Additionally, insulin resistance and/or diabetic states in human monocyte/macrophage-like THP-1 cells was shown to enhance histone acetylation in the gene body region of *TNFA* and *TNFA* gene expression (Honma et al., 2020). The small intestinal expression of genes related to carbohydrate digestion, such as *Si*, have been reported to be higher in insulin resistant OLETF rats than in non-insulin resistant OLETF rats (Adachi et al., 1999). The differences between white adipocytes and other tissues (liver, monocytic cells, and small intestine) can be caused by white adipocytes incorporating glucose in an insulin-dependent manner via SLC2A4 (generally called GLUT4). However, liver, monocyte/macrophage, and small intestine cells uptake glucose according to the blood glucose concentration in an insulin independent manner (Figure 5). Therefore, inactivation of gene body epigenetics via BRD4 and acetylation in white adipose tissue leads to activation of gene body epigenetics in the small intestine, liver and innate leukocytes. An imbalance can lead to the development and progression of type 2 diabetes.

A deficiency in *Brd4*, which is a BET family member and expressed primarily in white adipocytes, by gene targeting in mice was found to induce obesity (Wang et al., 2009). In contrast, *Brd4* heterozygous mice were found to be lean (Houzelstein et al., 2002). BRD2 inhibits white adipogenesis by repressing PPARγ2 and C/EBPα expression (Zang et al., 2013), whereas BRD4 enhances differentiation of white adipocytes by enhancing transcription elongation (Sakurai et al., 2017). Therefore, regulating the balance between BRD4 and BRD2 activities may inhibit metabolic diseases, such as obesity, type 2 diabetes, and related complications. Thus, the regulation of adipogenesis by BET family members including BRD4 and BRD2 should be examined in future research endeavors.

## Concluding Remarks

Acetylated histone and BRD4 gene body epigenetics regulate the expression of many carbohydrate-responsive metabolic genes in several metabolic organs. Insulin resistant and diabetic conditions induce the development of metabolic diseases by reducing the expression of BRD4-targeted carbohydrate-responsive metabolic genes in white adipose tissue and by inducing the expression of BRD4-targeted carbohydrate-responsive metabolic genes in the liver, small intestine, and innate leukocytes such as monocytes, macrophages, and neutrophils.
